# Single Chain Fragment Variable (scFv) Antibodies Targeting the Spike Protein of Porcine Epidemic Diarrhea Virus Provide Protection against Viral Infection in Piglets

**DOI:** 10.3390/v11010058

**Published:** 2019-01-14

**Authors:** Fanqing Zhang, Yuxue Chen, Yong Ke, Lei Zhang, Bo Zhang, Liang Yang, Jianguo Zhu

**Affiliations:** 1 Shanghai Key Laboratory of Veterinary Biotechnology, School of Agriculture and Biology, Shanghai JiaoTong University, Shanghai 200240, China; zero0fan@126.com (F.Z.); keyong@sjtu.edu.cn (Y.K.); barcelonazl@sjtu.edu.cn (L.Z.); zhangbo@126.com (B.Z.); 2 Shanghai Frontan Animal Health Corp., Shanghai 201502, China; xiaoxue@frontan.com (Y.C.); Yangliang@Frontan.com (L.Y.)

**Keywords:** porcine epidemic diarrhea virus, spike (S) protein, single chain fragment variable (scFv), protection

## Abstract

Porcine epidemic diarrhea virus (PEDV) is a highly contagious coronavirus that causes severe diarrhea and death in neonatal piglets. Passive immunization with neutralizing antibodies against PEDV is an effective prevention measure. In this study, single chain fragment variable (scFv) antibodies against PEDV were screened from the porcine scFv phage display library. After four rounds of biopanning, scFvs that showed higher affinity to the PEDV antigen were selected for further study. The scFv genes were cloned into the expression plasmid for recombinant protein expression. These scFvs were shown to inhibit PEDV infectivity by the plaque reduction neutralization assay. Immunofluorescence assay (IFA) revealed that the epitopes recognized by these scFvs were in the S1 region of the spike protein. The potential of scFvs to provide prevention against PEDV infections in piglets was further investigated. Piglets orally administered scFvs showed no to mild clinical symptoms, significantly less viral shedding, no mortality and no intestinal lesions. The field application also revealed that the survival rate of piglets was significantly increased by oral administration of scFvs. Our data support the potential role of scFvs in the prevention and treatment of PEDV infection.

## 1. Introduction

Porcine epidemic diarrhea (PED) is an acute, highly contagious enteric disease in pigs caused by porcine epidemic diarrhea virus (PEDV) [1]. The characteristic clinical signs of PEDV infection are vomiting, watery diarrhea, dehydration, and weight loss [2,3]. Pigs of all ages are susceptible to PEDV infection, and neonatal piglets under 2 weeks of age have the highest mortality rates, reaching 100% [2]. PEDV was initially reported in Europe in the early 1970s, and it has spread widely to multiple swine-producing countries [4]. Since 2010, the emergence of highly virulent PEDV strains has decimated swine farms in China [5]. These strains spread to the United States in 2013, wiping out more than 10% of the pig population of America in less than a year [6,7,8]. Since then, these new high-virulence PEDV strains have repeatedly emerged in many swine-raising countries, leading to devastating economic losses to the world swine industry [4,9]. The severity of these highly virulent variants emphasizes the importance of developing an effective method to control their spread and infection.

PEDV is a member of the *Alpha-coronavirus* genus, within the family *Coronaviridae* in the order *Nidovirales* [3,10]. PEDV possesses an ~28 kb single-stranded, positive-sense, RNA genome, which encodes seven open reading frames (ORF 1a/1b, and ORF 2-6) [11]. Among them, the first gene ORFs 1a/1b encode large replicase polyproteins, which are processed to generate 16 nonstructural proteins (nsp1-nsp16) [12]. ORF 2, –3, –4, –5, and –6 encode structural/accessory proteins, including spike (S) protein, nonstructural accessory protein, envelope (E) protein, membrane (M) protein, and nucleocapsid (N) protein, respectively [13].

The S protein is a type I glycoprotein that plays a crucial role in virus attachment, entry, receptor binding, cell membrane fusion and induction of neutralizing antibodies [14,15]. The S protein can be cleaved into S1 (residues 1–789) and S2 subunits (residues 790–1386) by host protease [16]. The S1 subunit contains the N-terminal domain (NTD, residues 1–233) that shows sialic acid binding activity and the C-terminal domain (CTD, residues 253–638) that attaches to the cell surface receptor (e.g., aminopeptidase N (APN)) [17]. The S2 subunit mediates virus–cell membrane fusion [16]. S protein is an excellent target for vaccine development for the induction of protective immunity against PEDV. Several studies have confirmed that antibodies, especially neutralizing antibodies stimulated by the vaccine expressing spike protein, are able to protect the host from PEDV infection [18,19]. Additionally, neutralizing antibodies against PEDV can be developed as candidates for passive protection. Lee et al. reported that egg yolk antibody (IgY) against S1 domain of spike protein efficiently protects neonatal piglets against PEDV, supporting the potential of antibody reagents as a prophylactic or therapeutic agent to protect piglets against PEDV infection [20].

Genetically engineered recombinant antibody fragments are increasingly being used in medical diagnosis and therapy in many diseases. The single chain fragment variable (ScFv), also called single-chain antibody, is one of the most popular types of genetically engineered antibodies [21,22]. The scFv consists of a variable light chain (V_L_) and heavy chain (V_H_) that are connected by a short peptide linker [23]. The advantages of scFv are its small size, low immunogenicity, high specificity, and ability to be genetically engineered. The scFv can be produced in bacterial expression systems for large-scale production. Although scFv is smaller than full-length IgG, it retains the complete antigen binding site [24]. Several scFvs have been produced to control virus infection, including scFvs against chicken infectious bursal disease virus, scFvs targeting human influenza virus H5N1, and scFvs against the phosphoprotein of Newcastle disease virus [25,26,27,28]. Thus, scFv is considered a potential reagent for the prevention and treatment of viral disease.

At present, there have been no reports of the selection of porcine scFvs to target the porcine pathogen. In this study, we constructed a scFv phage display library using peripheral blood lymphocytes of piglets induced with PEDV. The scFvs against PEDV were selected, and their neutralization efficiencies were evaluated. We further confirmed that the mechanism of scFvs neutralization of PEDV occurred through binding to the viral spike protein. The immunoprophylactic and therapeutic properties of scFvs in neonatal piglets against PEDV infection were further explored. Our results provide a foundation for the development of scFv-based drugs for the prevention and treatment of PEDV infection.

## 2. Materials and Methods

### 2.1. Ethics Statement

Animal experiments were performed in accordance with the recommendations laid out in the Guidelines for the Use of Laboratory Animals provided by the Science and Technology Commission of Shanghai Municipality (STCSM). The protocol was approved by the ethics committee of Shanghai JiaoTong University, School of Agriculture and Biology (approval number: 201600853).

### 2.2. Cells, Viruses and Plasmids

The Vero E6 cell line (ATCC^®^ CRL-1587^TM^) or HEK 293T cell line (ATCC^®^ CRL-3216^TM^) was cultured in Dulbecco’s Modified Eagle’s Essential Medium (DMEM) (Invitrogen, Carlsbad, CA, USA) supplemented with 10% fetal bovine serum (FBS; Gibco, Grand Island, NY, USA) and 100 μg/mL penicillin/streptomycin (Invitrogen). All cells were cultured at 37 °C in a 5% CO_2_ incubator.

The intestinal porcine epithelial cell line J2 (IPEC-J2) (kindly provided by Prof. Jianxiong Xv) was maintained in DMEM/F12 medium supplemented with 10% FBS and incubated in an atmosphere of 5% CO_2_ at 37 °C.

The PEDV (SH2012-5, GenBank accession number: MG837011.1) were propagated in Vero E6 cells in the presence of 5 µg/mL tosyl phenylalanyl chloromethyl ketone (TPCK)-treated trypsin (Worthington, Lakewood, NJ, USA).

To construct PEDV S1 and S2 domain-expressing plasmids, the PEDV S1 domain was amplified with the upstream primer containing *Eco*R I (underlined): TTGAATTCATGAAGTCTTTAACCTACTTC and the downstream primer containing *Xho* I (underlined): TTCTCGAGTCATTTACAACGAGAGTTACCATT. The PEDV S2 domain was amplified with the upstream primer containing *Eco*R I (underlined): TTGAATTCATGAGTCAAGATTGCACCCAC and the downstream primer containing *Xho* I (underlined): TTCTCGAGTCACTGCACGTGGACCTTTTC. PCR products were inserted into pCDNA3.1 (+) (Thermo Scientific Inc., Waltham, MA, USA) to form pCDNA3.1-PEDV-S1 and pCDNA3.1-PEDV-S2, respectively. The recombinant plasmids were verified by sequencing and indirect immunofluorescence assay (IFA).

### 2.3. Virus Purification by Sucrose Gradient Centrifugation

Confluent Vero E6 cell cultures infected with PEDV were harvested when 90% of the cells showed a viral cytopathic effect. Virus particles in the suspensions were purified by sucrose density gradient centrifugation as previously described [29]. The resulting band between the 40% and 60% sucrose solutions were collected with a syringe. After removing the sucrose, the presence of concentrated viral particles was determined by ELISA.

### 2.4. Molecular Cloning of scFv

A total of 20 PEDV serum-positive crossbreed (large white) pigs were purchased from a local breeding farm. Blood samples were collected from these pigs and used to isolate peripheral blood lymphocytes (PBLs). Total RNA was extracted from the PBLs using TRIzol reagent (Invitrogen) and used as template to synthesize full-length cDNA. The primers for the amplification of the heavy-chain variable regions (V_H_) and light-chain variable regions (V_L_) are listed in Table 1. In the first step of the PCR, the V_H_ gene and V_L_ genes (including V_Lκ_ and V_Lλ_) were amplified, respectively. In the second step of PCR, amplified V_L_ was added with a linker sequence to generate V_L_-Linker. Finally, V_H_ and V_L_-Linker were ligated together to form a full-length scFv by SOE-PCR.

### 2.5. Library Construction

The scFv PCR products were separated by electrophoresis and extracted from the gel using a commercial kit (Thermo Scientific Inc.). The purified fragments were inserted into the linearized pCANTAB5e vector (Biovector Inc., Beijing, China). The recombinant DNAs were transformed into *Escherichia coli* competent TG1 cells (Biovector) by electroporation (MicroPulser; Bio-Rad, Hercules, CA, USA) and grown on 2× YT-AG plates (100 µg/mL ampicillin and 2% glucose). The library size was calculated by counting the colonies of transformed bacteria. The recombinant phagemid transformed cells were then infected with M13KO7 helper phage to generate the rescued phage particles.

### 2.6. Biopanning

A 96-well ELISA plate was coated with 5 µg/mL purified PEDV particles at 4 °C overnight. After blocking with 5% non-fat milk in PBS, the rescued phages were added to each well. Unbound phages were removed by washing 10 times with PBST and 10 times with PBS. The PEDV-binding phages were eluted by the addition of 0.1 M glycine-HCl buffer (pH 2.2). Log phase-grown *E. coli* TG1 were infected with the eluted phages and then plated onto a 2× YT-AG plate. A total of four rounds of biopanning were performed.

### 2.7. Binding Affinity Analyzed by Phage ELISA

Phage ELISA was used to assess the phages that bound to PEDV. A 96-well plate was coated with 2 µg/mL of PEDV particles in 0.1 M NaHCO_3_ (pH 8.6), blocked with 5% nonfat milk, and then incubated with recombinant phage particles. After washing with PBST, 48 selected phages derived from the last round of biopanning were detected with HRP-conjugated anti-M13 monoclonal antibody (1:5000 dilution). The OD value at 450 nm was measured using a microplate reader (BioTek, Winooski, VT, USA).

### 2.8. Production of Soluble scFv

The scFv gene sequences of interest were subcloned into the pET-28(+) vector, and scFv expression was induced with 1.2 mM IPTG (Sigma-Aldrich, St. Louis, MO, USA). The bacterial cells were lysed by sonication and the scFv protein was purified using a Ni-NTA his-bind resin column (Merck, Madison, WI, USA) according to the manufacturer’s instructions. After washing the column two times with 20 mL of washing buffer (300 mM NaCl, 50 mM sodium phosphate buffer, 20 mM imidazole, pH 8.0), the scFv bound to the column was eluted with the elution buffer (300 mM NaCl, 50 mM sodium phosphate buffer, 100 mM imidazole, pH 8.0). The eluted scFv was assessed by 10% sodium dodecyl sulfate polyacrylamide gel electrophoresis (SDS-PAGE). For subsequent experiments, the eluted scFv was further dialyzed in 1000 mL PBS at 4 °C for 48 h, changing the PBS every 12 h. The purified scFv was concentrated using centrifugal filters with a 10-kDa cutoff (Merck), quantified using the bicinchoninic acid assay kit (Thermo Scientific Inc.) and stored at 4 °C.

### 2.9. Cytotoxicity Assay

The cytotoxic activity of scFv on Vero E6 and IPEC-J2 cells was determined using the 3-(4,5-dimethylthiazol-2-yl)-2,5-diphenyltetrazolium bromide (MTT) assay (Thermo Scientific Inc.). Briefly, approximately 2 × 10^4^ cells/well were seeded in a 96-well cell culture plate and cultured overnight. The next morning, scFv at two-fold serial dilutions (3.125–200 μg/mL) or PBS alone was added to each well. The cells were incubated for 48 h. Then, MTT solution (0.2 mg/mL) was added to each well, and the plate was further incubated at 37 °C for 4 h. The supernatant was then removed, and the colored formazan crystal in each well was dissolved by addition of 100 µL of SDS-HCl solution (Sigma-Aldrich). The OD value at 570 nm was measured using a microplate reader (BioTek). The cell survival rate was calculated as the (OD_570_ of scFv treatment)/(OD_570_ of PBS treatment) × 100%.

### 2.10. Neutralization Test

To determine whether scFv against PEDV possessed neutralization activity, a plaque reduction neutralization (PRN) assay was performed [30]. Briefly, purified scFv or combined cocktail (contains 2/3 mg/mL of each scFv) was adjusted to 2 mg/mL and filtered using a 0.22-µm membrane. Subsequently, scFv was serially diluted 2-fold and incubated with an equal volume of PEDV at a multiplicity of infection (MOI) of 0.01 for 1 h at 37 °C. Then, the scFv-virus mixture was inoculated onto a confluent monolayer of Vero E6 cells in 24-well plates and incubated at 37 °C for 1 h. A positive control (virus only, no scFv), a blank controls (no virus, no scFv) and a negative control (virus, non-related scFv) were also added to the well. The cells were overlaid with DMEM containing 1% low melting agarose (Sigma-Aldrich) and 5 µg/mL TPCK-treated trypsin. After 48–72 h of incubation, cells were fixed in 4% formaldehyde for 30 min and stained with 1% crystal violet solution for 20 min. The number of plaques was determined by manual counting and plaque reduction rate for each scFv was calculated using the formula: [1 − (average number of plaques for each dilution/average number of plaques in the positive control well)] × 100%. Neutralizing antibody titers were calculated as the reciprocal of the highest serum dilution that reduced the virus plaque count by 80% for scFv or combined cocktail. Each scFv or combined cocktail was tested in triplicate.

### 2.11. Indirect Immunofluorescence Assay (IFA)

Vero E6 cells or HEK 293T cells grown to 60% confluence on coverslips in six-well plates were transfected with pCDNA3.1-PEDV-S1/S2 or infected with PEDV at an MOI of 1. At 24 h post-infection or 24 h post-transfection, the cells were fixed in 4% paraformaldehyde, permeabilized with 0.5% Triton X-100 and blocked with 10% normal goat serum (Tiangen Biotech, Beijing, China) to block nonspecific binding sites. They were then incubated with 100 μL of purified scFv for 2 h. After three washes with PBS, anti-6 × His tag antibody (FITC) antibody (1:2000 dilution) (Abcam, Cambridge, MA, USA) was added. Images of the stained cells were visualized using a Nikon Eclipse 80i fluorescence microscope (Nikon, Sendai, Japan).

### 2.12. Epitope Analysis by Additive ELISA

To ascertain whether the scFvs recognized different epitopes on the PEDV antigen, an additive ELISA was performed to calculate the additivity index (AI). Briefly, 96-well plates were coated with PEDV antigen (10 μg/mL) and incubated overnight at 4 °C. 100 μg/mL of each scFv that was able to saturate the coated antigen was added alone or in combination. After incubation and washing, the bound scFvs were detected by the addition of HRP-conjugated anti-His mouse antibody (Yeasen, Shanghai, China). Normal mouse serum was used as a negative control. Competition for each scFv was determined by calculating the AI using the following equation: AI = [2A_1+2_/(A_1_ + A_2_) − 1] × 100%, where A_1_ and A_2_ represent the OD_450_ values for each of two scFvs tested, and A_1+2_ represents the OD_450_ value when the two scFvs are mixed. If the AI is above 50%, then the two scFvs recognize different antigenic epitope; if it is below 50%, then the two scFvs recognize the same antigenic epitope.

### 2.13. In Vitro Stability of scFv to Simulated Gastric Conditions

The stability of scFv to gastric conditions was evaluated using simulated gastric fluid (SGF). SGF contained 3.2 mg/mL pepsin (Sigma-Aldrich) in 0.03 M NaCl at pH 3 [31]. Purified scFv was added into 200 μL of SGF or PBS (control) and incubated at 37 °C with shaking for 0–3 h. Antibody activity was defined as the ability of scFv binding to PEDV and was assessed by ELISA. The result was expressed as relative activity (%) using the following formula: (OD_450_ value of scFv treated with SGF/OD_450_ value of scFv treated with PBS) × 100%.

### 2.14. In Vitro Stability of scFv to Simulated Intestinal Conditions

The stability of scFv to intestinal conditions was evaluated using simulated intestinal fluid (SIF). SIF contained 10 mg/mL pancreatin (Sigma-Aldrich) in 0.05 M KH_2_PO_4_ at pH 6.8 [31]. Purified scFv was added into SIF or PBS (control) and incubated at 37 °C with shaking for 0–4 h. The scFv activity was determined by ELISA. The result was expressed as relative activity (%) using the following formula: (OD_450_ value of scFv treated with SIF/ OD_450_ value of scFv treated with PBS) × 100%.

### 2.15. Immunohistochemistry

Two crossbred (large white) pigs were oral administrated with 50 mL of purified scFv using a flexible gavage feeding needle (Tianyuan Animal instrument Inc., Shandong, China). At 4 h post inoculation, pigs were humanely euthanized with 100% CO_2_. The section of jejunum was collected, fixed in 10% formalin for 24 h and immersed in 30% sucrose for 48 h. Tissue samples were then embedded in OCT compound (Sakura Finetek, Radnor, PA, USA) and sectioned at 10 µm thickness using a cryomicrotome (Leica CM1900; Wetzlar, Germany). Cryosectioned samples were transferred onto the polylysine pre-treated slides, stained with anti-His monoclonal antibody (Sigma-Aldrich) as primary antibody followed by FITC conjugated goat anti-mouse antibody as secondary antibody. The sections were omitted primary antibodies as negative control. The samples were visualized by a fluorescence microscopy (Nikon).

### 2.16. Animal Experiment

Crossbred (large white) piglets were obtained from PEDV seronegative pregnant sows at a local breeding farm after birth. The piglets were housed in separate sterile isolators and fed with commercial sterile milk and water. Experiment 1 (group 1 to 3) was assigned to measure the prophylactic efficiency of scFvs on the survival of piglets against PEDV challenge. Twelve 3-day-old piglets were randomly divided into three groups (*n* = 4). The scFvs were added to the milk to adjust the final concentration to 100 µg/mL. At 3 days of age, group 1 was orally administered 50 mL of the scFvs-milk mixture five times per day using a flexible gavage feeding needle (Tianyuan Animal Instrument Inc.); group 2 and group 3 were orally administered the PBS-milk mixture five times per day. On the next day, groups 1 and 2 were orally inoculated with 3 mL of DMEM containing 5 log_10_ PFU/piglet of PEDV. After the challenge exposure, groups 1 and 2 were orally administered the corresponding scFvs-milk or PBS-milk mixture for another two days. All animals were monitored for 10 days for the presence of clinical symptoms, including diarrhea, vomiting, anorexia and depression. The severity of clinical symptoms was evaluated. Fecal samples collected from the rectum using swabs were used to quantify viral shedding and evaluate the severity of diarrhea. The fecal consistency were scored as follows: 0, normal feces (no diarrhea); 1, pasty feces (mild diarrhea); 2, semiliquid feces (moderate diarrhea); and 3, liquid feces (severe diarrhea) [32]. Experiment 2 (group 4 to 6) was assigned to measure the prophylactic efficiency of scFvs on the small intestine of piglets against PEDV challenge. Nine 3-day-old piglets were randomly divided into three groups (*n* = 3). The treatment for piglets in groups 4 to 6 was the same as that for piglets in groups 1 to 3, respectively. At 5 days post challenge (dpc), piglets in groups 4 to 6 were humanely euthanized with 100% CO_2_ and subjected to pathological and histological examinations. After necropsy, lesions in the small intestine were observed. Small intestinal tissues were collected from each piglet and fixed in 10% formalin. The fixed specimen was trimmed, processed, embedded in paraffin, rehydrated and stained with hematoxylin and eosin (H&E). The stained sections were observed by light microscopy (Olympus, Tokyo, Japan).

### 2.17. qPCR Analysis

The fecal samples were homogenized in PBS, frozen and thawed 3 times, and they were centrifuged at 8000× *g* for 20 min. Total RNA was extracted from the supernatant, and cDNA was generated using a reverse transcription kit. The qPCR was performed in a 20-µL reaction volume containing 2 µL of template, 0.4 µL of forward (10 µM) and reverse primer (10 µM), 6.8 µL of H_2_O, 0.4 µL of ROX reference dye and 10 µL of SYBR Green qPCR premix (Clontech, Mountain View, CA, USA). The qPCR reaction was carried on an ABI 7300 Real-Time PCR System (Applied Biosystems, Foster, CA, USA) under the following conditions: an initial reverse transcription at 58 °C for 30 min, followed by an initial denaturation at 95 °C for 5 min, 40 cycles of 95 °C for 30 s, and 60 °C for 1 min. The primer sequences are listed in Table 1. The detection limit of the assay was determined by generating standard curves from serial 10-fold dilutions of known amounts of in vitro-transcribed RNA (1 × 10^12^) for the qPCR. The standard curve for PEDV qPCR has a slope of −3.462, indicating that the amplification efficiency was 94.47%. The detection limit was calculated as 1000 RNA copies per mL. The viral genome copy numbers per mL were determined, and each sample was assessed in triplicate.

### 2.18. Field Application

The field experiment was carried out in pig farms having outbreaks of diarrhea in winter. Fecal/intestinal samples were collected from piglets with watery diarrhea. Viral RNA was extracted and used as a template for RT-PCR on PEDV, transmissible gastroenteritis virus (TGEV) or porcine deltacoronavirus (PDCoV) [33,34,35]. Two farms that showed PEDV positive results were chosen for the application of scFvs. The scFvs were added to the milk to adjust the final concentration to 200 µg/mL. Piglets at two- to four-day-old that showed symptom of diarrhea were orally administrated with 40 mL of scFvs-milk three times per day. The other piglets inoculated with PBS-milk were served as negative control. The number of death and survival of piglets were recorded every day for five days. The mortality was calculated five days after first administration of scFvs.

### 2.19. Statistical Analysis

All the data were expressed as the means ± standard error of the mean (SEM). Data were compared for significance using a student’s t test or one-way ANOVA with the SAS 9.0 software package (SAS institute Inc., Campus Drive Cary, NC, USA). A *p*-value <0.05 was considered statistically significant.

## 3. Results

### 3.1. Construction of the scFv Library

Viral particles were purified by sucrose density gradient centrifugation and subjected to ELISA to confirm their immunogenicity. The results showed that purified PEDV could be used as antigen to screen PEDV-specific scFvs (Figure S1).

Antibody titers of the pigs that were immunized or infected with PEDV were evaluated by indirect ELISA (data not shown). RNA was extracted from PBLs and transcribed to the cDNA. Three rounds of PCR were performed to obtain the ~750 bp scFv gene fragment. (Figure S2a,b). The scFv fragments were then subcloned into linearized pCANTAB5e vector and confirmed by endonuclease digestion (Figure S2c). The recombinant plasmids were transformed into TG1 cells to construct the scFv library. The library size was determined to be 3.22 × 10^6^ cfu/mL by counting the colonies on plates. The library size was suitable for scFvs selection.

### 3.2. The scFv Phage Library Screening

Four rounds of biopanning were performed to screen PEDV-bound scFv from the scFv phage display using PEDV as bait. Compared with the 1^st^ round, the final output titers increased 72-fold to 3.54 × 10^7^ cfu/mL (Table 2). The output rates of the third and fourth rounds had the same order of magnitude, indicating that sufficient high-affinity antibodies could be screened after four rounds of consecutive biopanning.

### 3.3. The scFvs Screening by Indirect ELISA

**Figure 1:**
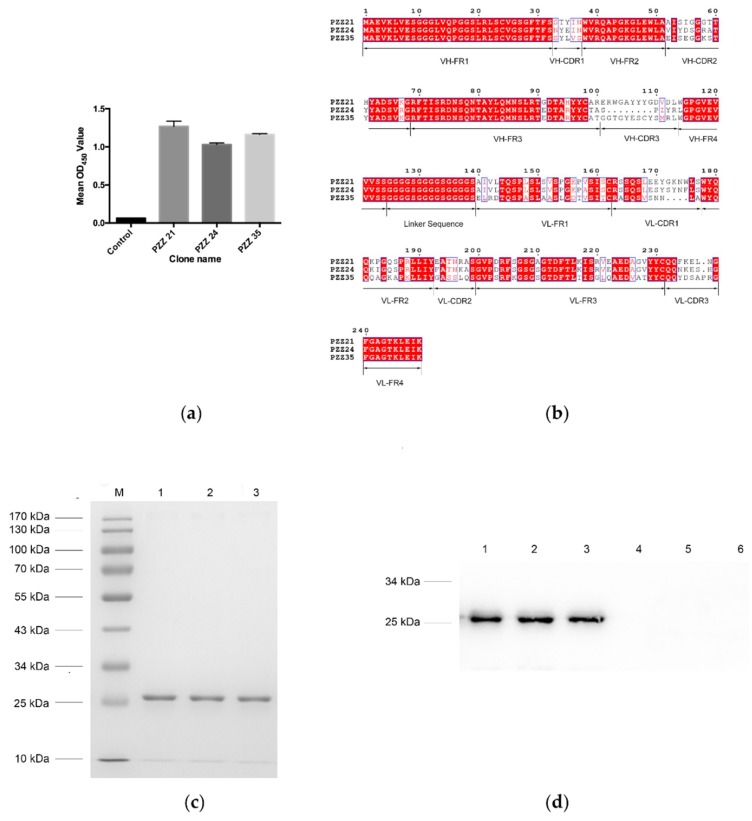

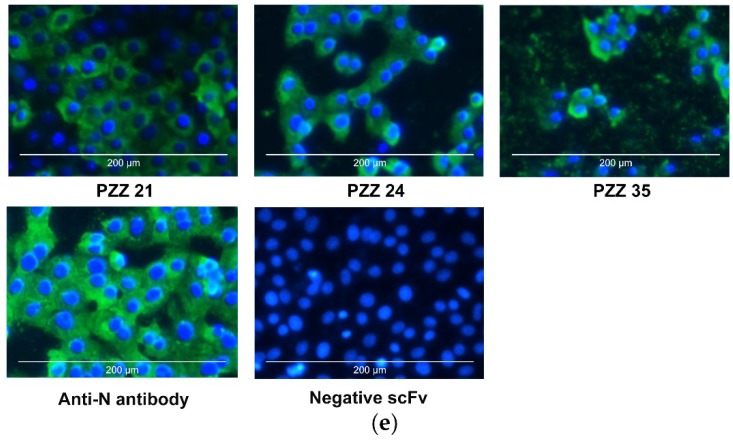
Identification of scFvs bound to porcine epidemic diarrhea virus (PEDV) antigen. (**a**) The single chain fragment variable (scFv) obtained from the biopanning process were further analyzed by phage ELISA. An scFv that showed no affinity to PEDV antigen was used as a negative control. Three scFvs with relatively higher values were subjected to further analysis. These scFvs were designated PZZ 21, PZZ 24, and PZZ 35, respectively. (**b**) The deduced amino acid sequences of PZZ 21, PZZ 24, and PZZ 35 were aligned using ClustalX (version 2.1, www.clustal.org/), and the figure was produced using ESpript (espript.ibcp.fr/). The dots (.) represent the missing amino acids. (**c**) The PZZ 21, PZZ 24, and PZZ 35 gene sequences were ligated into the expression vector, and the secreted recombinant antibody was purified using Ni-NTA resin and subjected to SDS-PAGE analysis. Lane M: protein molecular weight marker; Lane 1: purified PZZ 21; Lane 2: purified PZZ 24; Lane 3: purified PZZ 35. (**d**) Purified scFv PZZ 21, PZZ 24, and PZZ 35 were further confirmed by western blot analysis. A mouse anti-His monoclonal antibody was used as the primary antibody. Lane 1: PZZ 21 induced by IPTG; Lane 2: PZZ 24 induced by IPTG; Lane 3: PZZ 35 induced by IPTG; Lane 4: PZZ 21 not induced by IPTG; Lane 1: PZZ 24 not induced by IPTG; Lane 6: PZZ 35 not induced by IPTG. (**e**) PZZ 21, PZZ 24, and PZZ 35 bound to PEDV-infected cells. PEDV-infected Vero E6 cells were incubated with PZZ 21, PZZ 24, or PZZ 35 at 24 h post-infection and then stained with the FITC conjugated anti-His monoclonal antibody. PEDV-infected cells stained with anti-N PEDV polyclonal antibody were used as the positive control. Stained cells were observed under a fluorescence microscope.

To obtain specific and high-affinity clones against PEDV, phage ELISA was performed. Forty-eight clones were randomly picked from the final round phage display library, and 22 positive PEDV-binding clones were obtained (OD_450_ value of sample/negative > 2). Among these positive phage clones, three scFvs showing relatively higher OD values were selected for further study (Figure 1a). These scFvs were designated PZZ 21, PZZ 24 and PZZ 35, respectively.

### 3.4. DNA Sequence Analysis of Positive scFv

DNA sequencing of PZZ 21, PZZ 24 and PZZ 35 confirmed that V_H_ gene fragments were connected to V_L_ gene fragments by a linker. The deduced amino acid sequences of positive clones were aligned using clustalW2 software (http://www.ebi.ac.uk/Tools/msa/clustalw2/). Amino acid sequence analysis using IMGT software (http://www.imgt.org/IMGT_vquest/vquest) revealed that both porcine scFvs belonged to immunoglobulin V_H_ for the heavy chain. PZZ 21 and PZZ 24 had V_Lκ1_ for the light chain, and PZZ 35 had V_Lκ3_ for the light chain. Alignment of the CDR sequences revealed a high variability in both heavy and light variable chains, especially in V_H_-CDR3 and V_L_-CDR3 (Figure 1b).

### 3.5. Identification and Expression of scFv Bound to PEDV

Since the pET-28a(+) vector contains (His)_6_-tags at both N- and C-termini, these scFvs were further purified using Ni-NTA His binding resin. SDS-PAGE indicated that the scFvs were successfully purified in a soluble and correct folding form (Figure 1c). The molecular weight of the purified protein was ~28 kDa, which corresponded to the predicted size. Purified scFv protein was also subjected to western blotting. The results showed that purified scFvs reacted with mouse anti-His tag antibody (Figure 1d). To confirm whether purified scFv specifically bound to PEDV, an IFA assay was performed. The result showed that PZZ 21, PZZ 24 and PZZ 35 stained PEDV-infected Vero E6 cells but did not react with uninfected control cells, indicating that purified scFv specifically bound to PEDV (Figure 1e).

### 3.6. Evaluation of the In Vitro Cytotoxicity of scFv

The MTT assay was performed to investigate the possible cytotoxicity of PZZ 21, PZZ 24, or PZZ 35 toward Vero E6 cell growth. As shown in Figure S3, different concentrations of scFv (3.125 μg/mL–200 μg/mL) showed no effect on Vero E6 cell proliferation. Additionally, PZZ 21, PZZ 24, and PZZ 35 showed no cytotoxic effect toward the porcine IPEC-J2 cell line, suggesting that scFv was also safe for intestinal porcine epithelial cells (Figure S3).

### 3.7. Neutralization Effect of scFv to PEDV

To determine whether the three scFvs neutralized PEDV infection, a PRN assay was performed. A scFv that showed no affinity to PEDV by ELISA was used as a negative control. Two serial dilutions of the scFvs working stocks (50–0.39 μg/mL) were tested in triplicate. As shown in Table 3, virus neutralization titer of PZZ 21, PZZ 24, and PZZ 35 were 6.25 μg/mL, 6.25 μg/mL and 12.5 μg/mL, respectively (Table 3). No neutralizing activity was observed for the negative control scFv. These results demonstrated that scFvs have the potential to neutralize viral infection in vitro.

We mixed three scFvs in a cocktail (2/3 mg/mL of each scFv) to investigate whether the combination of these scFvs might have a better effect on neutralizing virus infection than when used alone. The results showed that a cocktail of these scFvs neutralized viral infection at 3.125 μg/mL, demonstrating a superior effect than the use of scFv alone (Table 3). A cocktail of three scFvs was further used in the animal experiment.

### 3.8. Identification of the Protein Recognized by scFv Using IFA

**Figure 2:**
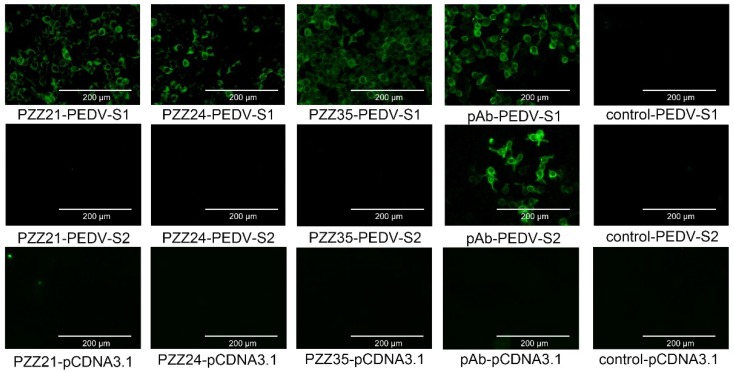
The scFv binds to the spike (S) protein in PEDV-infected cells. Identification of the viral protein recognized scFv by immunofluorescence assay (IFA). Recombinant plasmids pCDNA3.1-PEDV-S1 or pCDNA3.1-PEDV-S2 were transfected into cells. PZZ 21, PZZ 24, or PZZ 35 was added to confirm its reaction with the S1 or S2 domain. Mouse anti-PEDV polyclonal antibody was used as a positive control.

The PEDV spike protein is the only known target of PEDV-neutralizing antibodies. Since PZZ 21, PZZ 24, and PZZ 35 showed neutralizing effects on PEDV infection, we further clarified whether these scFvs could specifically recognize the spike protein. Our lab has previously constructed the eukaryotic expressing vector harboring full-length spike protein (pCDNA3.1-PEDV-S). However, pCDNA3.1-PEDV-S transfected cells showed weak fluorescence, as observed by IFA. To solve this problem, we constructed pCDNA3.1-PEDV-S1 and pCDNA3.1-PEDV-S2 expressing the S1 domain and S2 domain of spike protein, respectively. The expression of these plasmids was confirmed by the IFA assay using anti-PEDV polyclonal antibody. As shown in Figure 2, all scFvs reacted with S1 protein but not with S2 protein or pCDNA3.1 (+) empty vector. These results indicated that PZZ 21, PZZ 24, and PZZ 35 specifically bound to the S1 domain of S protein to neutralize viral infection (Figure 2).

### 3.9. Additive ELISA

To compare the recognized antigenic epitopes on PEDV antigen by scFvs, additive ELISA was performed to calculate the AI values. The AI values resulting from the combination of PZZ 21 with PZZ 24 and PZZ 35 were 73.8% and 82.5%, respectively. The AI value of PZZ 24 with PZZ 35 was 78.4%. These results confirmed that PZZ 21, PZZ 24, and PZZ 35 recognize different epitopes on the PEDV antigen (Table 4).

### 3.10. Effect of SGF or SIF on the Stability of the scFv

In order to evaluate the successful delivery to the small intestine, pigs were orally administrated with purified scFvs and the sections of jejunum were collected. The immunohistochemistry was performed using anti-His tag monoclonal antibody and FITC conjugated goat anti-mouse antibody. We observed signal of scFvs at the villi, lamina propria and muscular propria (Figure S4a). These data suggest that the purified scFvs were successfully delivered to the small intestine.

The effect of gastric conditions on scFv was evaluated using SGF. At intervals of 0, 0.5, 1, 2, and 3 h, samples were collected and neutralized with 200 mM Na_2_CO_3_ to stop proteolytic reaction. Indirect ELISA was performed to analyze the scFv activity after treatment with SGF. Results showed that PZZ 21, PZZ 24 and PZZ 35 maintain 82%, 72% and 66% activity at 1 h post-incubation with SGF, respectively. Even at 3 h post-incubation with SGF, scFv activity was still detectable in the SGF (Figure S4b).

The effect of small intestional conditions on scFv was evaluated using SIF. At intervals of 0, 1, 2, 3, and 4 h, samples were collected and stored at 4 °C to stop the reaction. Each scFv activity after SIF treatment was assessed by ELISA. Results showed that scFv was not significantly affected by incubation in SIF for 1 h (*p* < 0.01). The activity of each scFv retained 70–80% antibody activity after 4 h incubation with SIF (Figure S4c).

### 3.11. Prophylactic Efficiency of scFvs in Piglets

**Figure 3:**
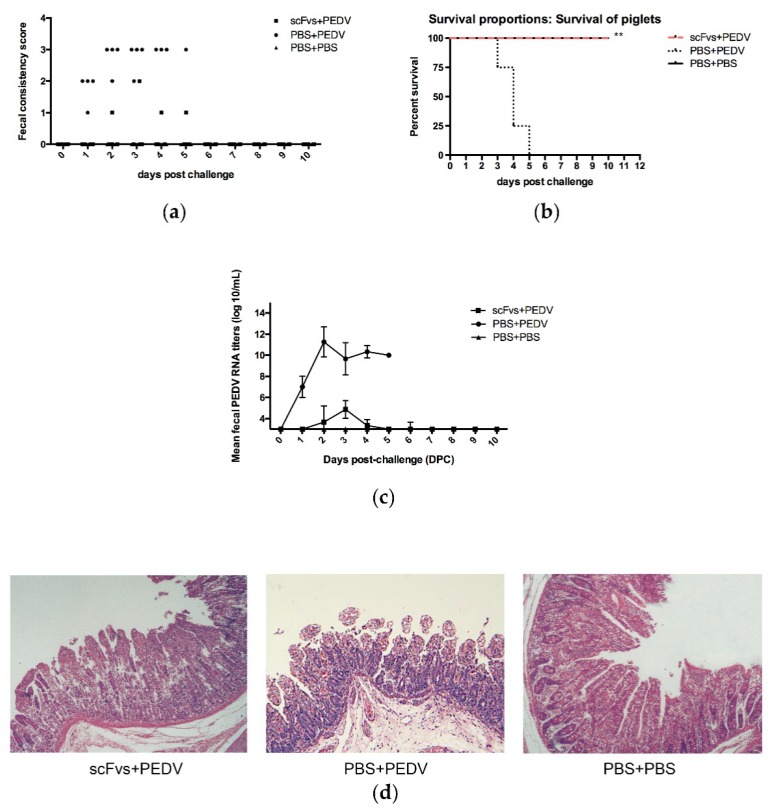
Evaluation of the prophylactic efficiency of scFvs in piglets challenged with prevalent PEDV. (**a**) After challenge with PEDV, the fecal consistency scores were recorded daily. Each dot indicates the score of an individual piglet at different days post-challenge (dpc). The fecal consistency was scored as follows: 0, normal feces; 1, pasty feces; 2, semiliquid feces; 3, liquid feces. (**b**) Piglets orally administered scFvs survived after PEDV infection. Animal survival was observed for 10 dpc. The log-rank (Mantel–Cox) test was used to calculate statistical differences between each group, and *p* < 0.01 was considered a significant difference. (**c**) Fecal samples were collected using cotton swabs and used to extract viral RNA. Quantitative real-time PCR was performed using primers targeting the PEDV N gene. The detection limit was 3 log10 copies/mL, and values lower than the detection limit were considered to denote negative samples. (**d**) Small intestinal tissues were collected from piglets at 5 dpi. The tissues were fixed in 10% formalin, embedded in paraffin and stained with hematoxylin and eosin (H&E). The stained sections were observed by light microscopy at 400× magnification.

Passive immunization with antibodies is an effective method for protecting piglets against PEDV infection. To confirm the prophylactic efficiency of scFvs, piglets were orally administered a cocktail of three scFvs and challenged with the prevalent PEDV strain. Clinical signs observed during the challenge phase are listed in Table 5. After challenge, piglets (4/4) in group 2 showed diarrhea at 1 day dpc. At 2–4 dpc, all the piglets in group 2 developed severe diarrhea, and no piglets in group 2 recovered from the diarrhea (Figure 3a). Comparatively, only one piglet (1/4) in group 1 showed mild to moderate diarrhea, the onset of diarrhea was delayed to 2 dpc, and the piglets recovered from the diarrhea at 6 dpc (Figure 3a). In addition to diarrhea, the piglets in group 2 also showed other clinical signs, including loss of appetite and mental depression (see Table 6). No mortality was observed in groups 1 and 3, whereas 100% (4/4) of the piglets in group 2 died 3–5 days post-infection (see Table 5 and Figure 3b).

Viral shedding of PEDV was quantified by real-time PCR based on the detection of the PEDV N gene. Rectal samples were collected from piglets every day after virus challenge. In group 2, piglets start to shed virus into the stool at 1 dpc, and the peak viral RNA titer was 11.27 ± 1.42 log10 copies/mL at 2 dpc (Figure 3c). In group 1, PEDV virus started to be detected at 2 dpc and continued for seven days, and the highest fecal PEDV RNA titer was significantly lower than in group 2 (*p <* 0.01) (see Table 5 and Figure 3c). These results indicated that scFv treatment could inhibit viral replication and shedding from PEDV-infected piglets.

All the surviving piglets in group 4 to 6 were humanely euthanized at 5 dpc. At necropsy, group 5 showed typical viral enteritis, including thin and transparent walls of the small intestine, and fluid filling the small intestine. No lesions were observed in the small intestine of groups 4 and 6 (data not shown). Histological examination revealed that the length of the jejunum villi was shortened, and severe fracture of the small intestinal mucosa, necrosis, villous atrophy and extensive intestinal epithelial degeneration were observed in group 5 (Figure 3d). In groups 4 and 6, the acinar structure of a small portion of the intestine remained intact (Figure 3d). Taken together, these results indicated that scFvs could provide protection to piglets against PEDV infection.

### 3.12. Field Application of scFvs

Forty-four samples from piglets with watery diarrhea and dehydration were subjected to PEDV, TGEV or PDcoV diagnosis. As shown in Table S1, 36 samples (81.8%) were PEDV positive and 0 samples were TGEV or PDcoV positive. Then, the therapeutic efficiency of scFvs was tested in these farms showing PEDV positive results. Part of the piglets from the same litters was orally administrated with scFvs-milk mixture everyday. The other piglets from the same litters were inoculated with PBS-milk. The survival rate at 5 days after inoculation was increased significantly in scFvs-milk treated piglets (48.4%) compared with the control group (8.6%) (Table 7). These results indicated that piglets were protected from PEDV infection by scFvs.

## 4. Discussion

In 2010, new variants of PEDV that emerged in China had a high morbidity (90–100%) and mortality (70–100%) in suckling piglets. The emergent PEDV strains are genetically distinct from the classical vaccine CV777 strain [13,36]. They are characterized by deletions, insertions or amino acid substitutions in the S gene, compared to the CV777 strains [4,6,37]. Even though many swine populations have been immunized with the classical vaccine CV777, it could only provide limited cross-neutralizing activity in swine populations against new variant strains, thus leading to immunity failure [38]. The strain used in the present study was isolated from piglets showing typical PED clinical symptoms. It belongs to the genotype of new variant strains, representing epidemic and pandemic field strains in China. We used this strain as an antigen to select specific porcine scFvs that could neutralize virus infection, and the prophylactic efficiency of scFvs was also evaluated.

The traditional strategy to protect piglets from PEDV infection is to immunize pregnant sows with inactivated or attenuated vaccines [39]. Neutralizing antibodies are produced in the colostrum or milk of sows, and newborn piglets are passively protected from PEDV when they suckle immune dams (lactogenic immunity) [3,40]. Although these vaccines are widely used and considered to be effective, not all sows develop solid lactogenic immunity due to the immunization route of the vaccine, and sucking pigs are still vulnerable to infection unless they obtain adequate maternal antibodies from the milk [19,41]. Additionally, this method does not provide protection to postweaned piglets. PEDV-specific antibodies are rapidly lost in postweaned piglets, and they become susceptible to PEDV. Moreover, amino acid sequence mutations in the new variant strains will cause immunization failure of commercial vaccines [42]. Based on these aspects, feeding foreign antibodies (passive immunization) to piglets is more suitable than vaccination to prevent neonatal diarrheal diseases. Egg yolk immunoglobulin (IgY) from chickens immunized with PEDV antigen has been administered to piglets, and the results showed that IgY is able to reduce mortality in piglets after challenge exposures [20]. Although IgY prepared from immunized chickens has the characteristics of high affinity, a large yield, and a simple isolation process, the production cost of high-quality IgY for large-scale applications still remains higher than that of commercial antibiotics or vaccine [43]. Since IgY is of chicken origin, it might cause immunogenic side effects in the host.

In this study, scFvs was prepared from pigs immunized or infected with PEDV. Since the host immune system was stimulated by the specific pathogen in PEDV-induced pigs, the abundant and mature antibodies were biased against PEDV [44]. After four rounds of biopanning, three scFvs with high affinity to PEDV were successfully screened. The predicted amino acid sequences of these two scFvs were aligned and analyzed. The results showed that the variation in the complementary determining regions (CDRs) were significantly greater than in the framework regions (FRs). The lengths of CDR3 in the V_L_ and V_H_ were not equal among the three scFvs, indicating the presence of amino acid insertions or deletions in these regions. Since CDRs contribute to the specificity of binding to a specific epitope, differences in the diversity of CDRs in the V_L_ and V_H_ domains may reflect the different binding epitopes of these scFvs.

The synergistic and/or additive effects of a cocktail of neutralizing antibodies that recognize different epitopes have been reported in numerous studies on different viruses [45]. According to the “occupancy” model (multihit theory) of neutralization, procuring a sufficient antibody density on the surface of a viral pathogen is a crucial and pivotal factor in viral neutralization along with recognition of the neutralizing epitopes [46]. This procedure may not be feasible in many cases where a single epitope is recognized, but it can be achieved when more than one epitope is recognized on the viral surface. In this study, we mixed three scFv in a cocktail to investigate whether the combination of these scFvs would have a better effect on the neutralization of viral infection compared with their use alone. The results showed that virus neutralization titer of a cocktail of scFvs was 3.125 μg/mL and neutralization titer of the individual scFv was 6.25 μg/mL or 12.5 μg/mL. Thus, a cocktail of three scFvs was used in the animal experiment.

Most monoclonal antibodies or scFvs that demonstrate a neutralization effect target viral surface glycoproteins owing to their important role in viral adherence to host cell receptors. We analyzed whether PZZ 21, PZZ 24 and PZZ 35 bind to PEDV spike protein to inhibit viral infection. The IFA assay and additive ELISA confirmed that all three scFvs reacted with different epitopes of S1 the domain but not the S2 domain. Previous reports have shown that the main neutralizing epitopes of S protein are located in the S1 domain [47,48], and two neutralizing domains have been identified in the S1 region, including NTD/S0 [49], COE (aa 499–636) [50], and S1D (aa 636–789) [48]. The exact epitopes recognized by these scFvs need further elucidation by pepscan ELISAs. Previous studies have reported that the major genetic variations of PEDV are concentrated in the S1 portion of the S gene, especially in the N-terminus of the S1 gene [51]. Even a single amino acid mutation in this region can change the structure of the spike protein, which may explain why current vaccine strains fail to protect piglets from new emerging variant strains [52]. Our study has confirmed that these scFvs neutralize PEDV by targeting to the S1 domain. Whether these three scFvs also influence PEDV CV777 replication, or just specifically recognize unique epitope on the variant strain, this still need further study.

After confirming that scFvs could neutralize the PEDV infection in Vero E6 cells, we subsequently tested the prophylactic efficacy of scFvs in neonatal piglets. Piglets inoculated with a cocktail of scFvs survived after challenge with virulent PEDV, and piglets treated with PBS followed by PEDV infection showed 100% mortality, suggesting that scFvs provides effective passive immunity to viral infection. Piglets treated with scFvs showed no to moderate diarrhea, and the intestinal mucosa remained intact under histological examination, indicating the scFvs could block the virus from invading the small intestine. The effect of scFvs is slightly influenced by the low pH and enzymatic degradation in the gastrointestinal (GI) tract after oral administration. To solve these problems, biodegradable polymeric nanoparticulates such as chitosan and its derivatives that can protect proteins from degradation may be a prospective strategy for oral delivery [53]. Previous studies have demonstrated the possibility of chitosan-based nanoparticle as carriers for the oral delivery of antimicrobial peptide [54], bacteriophages [55], antigens [56] and small interfering RNAs (siRNAs) [57]. Currently, we are focusing on the selection of suitable chitosan derivatives to protect scFvs from enzymatic degradation and modulate scFvs pharmacokinetics to improve their efficacy.

In summary, three scFvs specifically targeting PEDV were generated from porcine PBLs. These scFvs showed nontoxicity in vitro and could neutralize PEDV infection by binding to different epitopes of the spike protein. Animal experiments confirmed the protective efficiency of scFvs in piglets against PEDV challenge. Field application demonstrated that scFvs oral administration could reduce the mortality of piglets. To the best of our knowledge, this is the first report to examine the antiviral activity of porcine origin scFvs against PEDV. Our results provide a basis for the development of scFv-based drugs for the treatment and prevention of PEDV-induced diarrhea.

## Figures and Tables

**Table 1:** List of primers for construction of the porcine antibody library.

Primer	Sequence (5′-3′)
V_H_-backward	ATGGCCGAGGWGAAGCTGGTGGAGTCYGG
V_H_-forward	ACTCGAGACGACGACTTCAACGCCTGG
V_Lκ1_-backward	GCCATYGTGCTGACCCAGASTCC
V_Lκ2_-backward	GAGACTCGTSATGACCCAGTCTCC
V_Lκ3_-backward	GAGCTGCGTGATACACAGTCTCC
V_Lκ_-forward	CGTTTGAKYTCCAGCTTGGTCCC
V_Lλ_-backward	CAGRCTGTGGTGACVCAGGAGCC
V_Lλ_-forward	ACCGAGGACGGTCAGCTGGGTGC
V_Lκ1_-backward-Linker	GGTGGCCTCGAGTGGTGGCGGTGGCTCGGGCGGTGGTGGATCCGGTGGCGGCGGGTCTGCCATYGTGCTGACCCAGASTCC
V_Lκ2_-backward-Linker	GGTGGCCTCGAGTGGTGGCGGTGGCTCGGGCGGTGGTGGATCCGGTGGCGGCGGGTCTGAGACTCGTSATGACCCAGTCTCC
V_Lκ3_-backward-Linker	GGTGGCCTCGAGTGGTGGCGGTGGCTCGGGCGGTGGTGGATCCGGTGGCGGCGGGTCTGAGCTGCGTGATACACAGTCTCC
V_Lλ_-backward-Linker	GGTGGCCTCGAGTGGTGGCGGTGGCTCGGGCGGTGGTGGATCCGGTGGCGGCGGGTCTCAGRCTGTGGTGACVCAGGAGCC
V_H_-backward (*Sfi* I)	GC*GGCCCAGCCGGCC*ATGGCCGAGGWGAAGCTGGTGGAGTCYGG
V_Lκ_-forward (*Not* I)	TT*GCGGCCGC*ACGTTTGAKYTCCAGCTTGGTCCC
V_Lλ_-forward (*Not* I)	TT*GCGGCCGC*ACCGAGGACGGTCAGCTGGGTGC
PEDV-N-F	ACCTCCTACTTCACGTGCAA
PEDV-N-R	GTGATGTCATTCCACCACGG

The restriction sites of *Sfi* I and *Not* I are shown in italic. The linker sequence is underlined.

**Table 2:** Input and output numbers of the phage library from four rounds of biopanning.

Round of Screening	Input (cfu/mL)	Output (cfu/mL)	Output/Input (%) ^a^	Enrichment Fold	Total Enrichment Fold
1st	1.62 × 10^12^	6.67 × 10^5^	4.11 × 10^−7^	1	
2nd	1.57 × 10^12^	2.72 × 10^6^	1.73 × 10^−6^	4.2	
3rd	2.18 × 10^12^	3.22 × 10^7^	1.48 × 10^−5^	8.6	
4th	1.19 × 10^12^	3.54 × 10^7^	2.97 × 10^−5^	2.0	72.4

^a^ Output/Input (%) = (Output number × 100)/(Input number).

**Table 3:** Summary of neutralizing activity of scFv against PEDV.

scFv	VN Titers ^a^
PZZ 21	6.25 μg/mL
PZZ 24	6.25 μg/mL
PZZ 35	12.5 μg/mL
Combined cocktail	3.125 μg/mL

^a^ An 80% reduction in the number of plaques was used as a cutoff to determine neutralizing antibody titers.

**Table 4:** Additivity index (AI) measure by the additive ELISA.

scFv	PZZ 21	PZZ 24	PZZ 35
	AI % ^a^	AI %	AI %
PZZ 21	-	73.8	82.5
PZZ 24	73.8	-	78.4
PZZ 35	82.5	78.4	-

^a^ AI was calculated using the equation: AI = [2A_1+2_/(A_1_ + A_2_) − 1] × 100%, where A_1_ and A_2_ represent the OD_450_ values for each of two scFvs tested, and A_1+2_ represents the OD_450_ value when the two scFvs are mixed.

**Table 5:** Summary of piglets orally administered scFvs and challenged with virulent PEDV.

Treatment	Mortality Rate (%) ^a^	Diarrhea Rate (%) ^a^	Onset of Diarrhea (dpc)	Onset of Fecal RNA Shedding (dpc)	Peak Fecal RNA Shedding Titers (log10 copies/mL) ^a^
Group1: scFv + PEDV (*n* = 4)	0 (0/4) A	25 (1/4) A	3	2	4.87 ± 0.85 B
Group2: PBS + PEDV (*n* = 4)	100 (4/4) B	100 (4/4) B	1	1	11.27 ± 1.42 A
Group3: PBS + PBS (*n* = 4)	0 (0/4) A	0 (0/4) A	ND	ND	ND

^a^ Different uppercase letters indicate a statistically significant difference between groups (*p* < 0.01). ND: Not detected.

**Table 6:** Clinical signs of piglets orally administered scFvs and challenged with virulent PEDV.

Treatment	Days Post PEDV Challenge									
	1	2	3	4	5	6	7	8	9	10
scFv + PEDV (*n* = 4)	− ^a^/− ^b^	− ^a^/− ^b^	− ^a^/− ^b^	− ^a^/− ^b^	− ^a^/− ^b^	− ^a^/− ^b^	− ^a^/− ^b^	− ^a^/− ^b^	− ^a^/− ^b^	− ^a^/− ^b^
PBS + PEDV (*n* = 4)	− ^a^/− ^b^	+ ^a^/+ ^b^	+++ ^a^/+ ^b^	+++ ^a^/+ ^b^	+++ ^a^/+ ^b^	ND	ND	ND	ND	ND
PBS + PBS (*n* = 4)	− ^a^/− ^b^	− ^a^/− ^b^	− ^a^/− ^b^	− ^a^/− ^b^	− ^a^/− ^b^	− ^a^/− ^b^	− ^a^/− ^b^	− ^a^/− ^b^	− ^a^/− ^b^	− ^a^/− ^b^

ND: Not detected due to death of the piglets. ^a^: Degree of appetite loss: − normal appetite; + slight loss of appetite; ++ moderate loss of appetite; +++ no appetite. ^b^: Mental depression: − normal; + depression.

**Table 7:** Evaluation of therapeutic efficacy of scFvs in the pig farms.

Farms	Survival Piglets/Total Piglets ^a^		Litters
	scFv-Milk	PBS-Milk	
A	21/41	2/23	6
B	9/21	1/12	3
Sum	30/62 (48.4%) A	3/35 (8.6%) B	9

^a^ Different uppercase letters indicate a statistically significant difference between groups (*p* < 0.01).

## Supplementary Materials

Supplementary materials can be found at http://www.mdpi.com/1999-4915/11/1/58/s1.
