# Supplementary material for: Single Chain Fragment Variable (scFv) Antibodies Targeting the Spike Protein of Porcine Epidemic Diarrhea Virus Provide Protection against Viral Infection in Piglets
Source: Viruses. 2019 Jan 14;11(1):58. doi: 10.3390/v11010058 (PMC6356844; doi:10.3390/v11010058)
Supplement: Supplementary file 1 [file viruses-11-00058-s001.pdf]

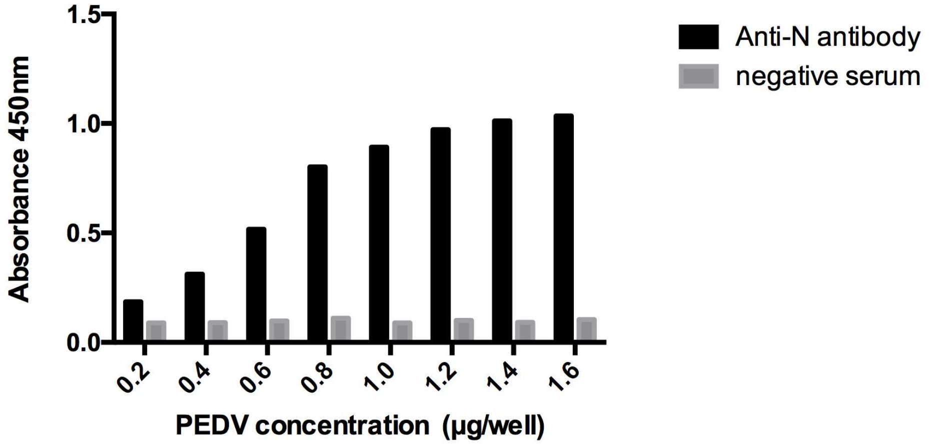

Figure S1. Purified PEDV reacted with anti-nucleocapsid (N) polyclonal antibody in a dose-dependent manner by ELISA. Purified PEDV were coated onto ELISA plates at a concentration from 0.2 μg/well to 1.6 μg/well. Mouse anti-N polyclonal antibody was used as the primary antibody (1:3000 dilution), and HRP-conjugated anti-mouse antibody was used as the secondary antibody. Unimmunized mouse serum was used as negative serum.

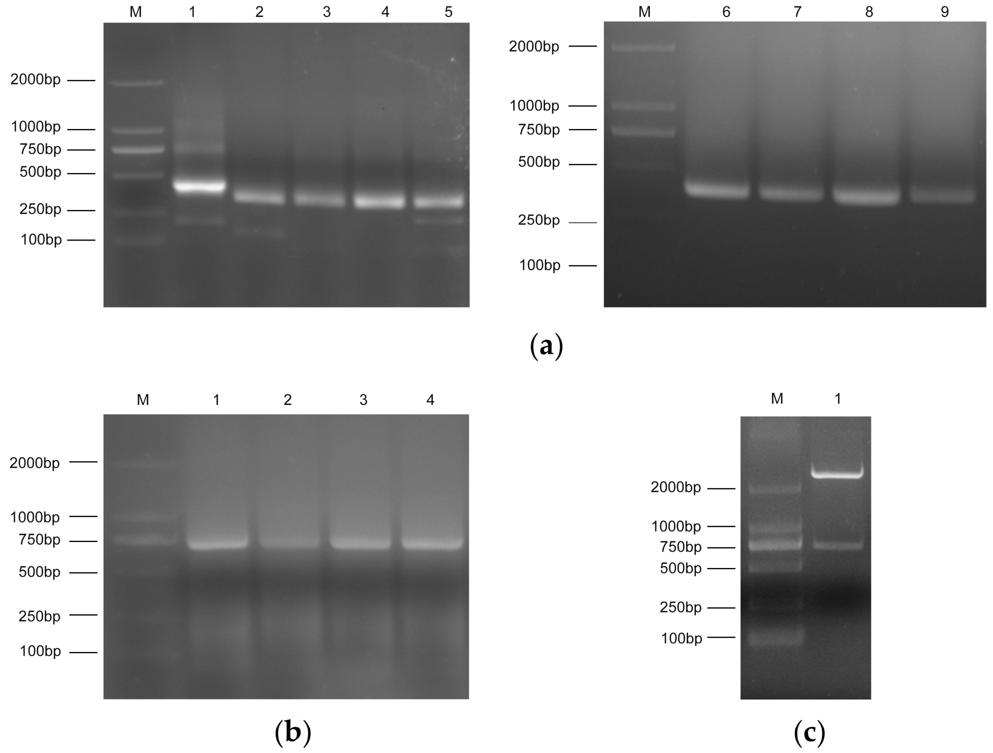

Figure S2. Construction of the anti-PEDV scFv antibody phage library. Three rounds of PCR were performed to obtain full-length scFv fragments. (**a**) In the first round, V_H and V_L genes were amplified and analyzed by agarose gel electrophoresis. Lane M, 2000-bp DNA ladder marker

(Takara); Lane 1. V_H PCR product; Lane 2-5, $V_{L\kappa1}$, $VL_{\kappa2}$, $V_{L\kappa3}$ and $V_{L\lambda}$ PCR product, respectively. In the second round, V_L fragments were fused to linker sequence by PCR. Lane M, 2000-bp DNA ladder marker (Takara); Lane 6-9, linker-$V_{L\kappa1}$, linker-$V_{L\kappa2}$, linker-$V_{L\kappa3}$) and linker-$V_{L\lambda}$, respectively. (**b**) In the final round, scFv fragments were obtained by SOE-PCR. Lane M, 2000-bp DNA ladder marker (Takara); lane 1-4, scFv 1 (VH-linker-$V_{L\kappa1}$), scFv 2 (VH-linker-$V_{L\kappa2}$), scFv 3 (VH-linker-$V_{L\kappa3}$) and scFv 4 (VH-linker-$V_{L\lambda}$), respectively. (**c**) The ligation of scFv with phagemid pCANTAB5e was confirmed by endonuclease digestion. Lane M, 5000-bp DNA ladder marker; lane 1, recombinant phagemid was double digested with enzyme *Not* I and *sfi* I.

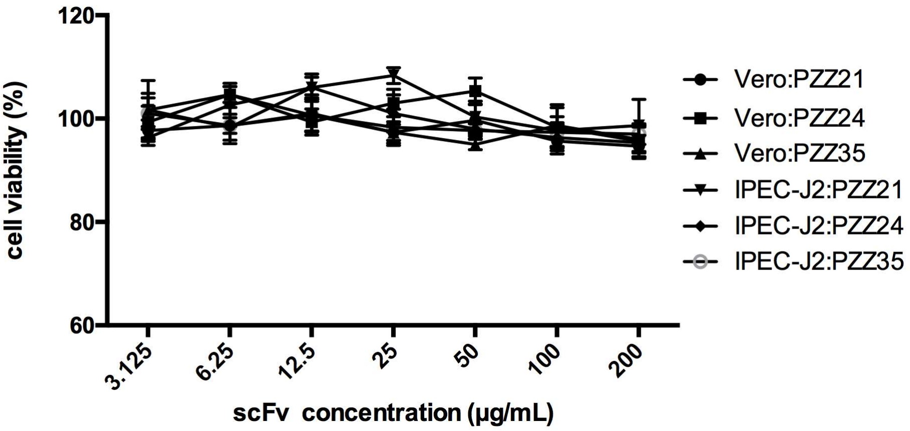

Figure S3. Viability of the cells after treatment with different concentrations of purified scFv by the MTT assay. Vero E6 cells or porcine IPEC-J2 cells were incubated with 2-fold diluted purified scFv (3.125 µg/mL-200 µg/mL), and the cell viability was calculated using the MTT assay kit. PBS treatment was used as a negative control.

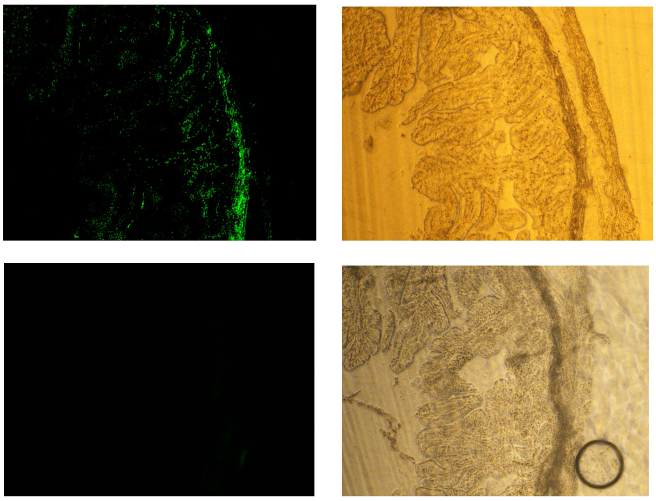

(a)

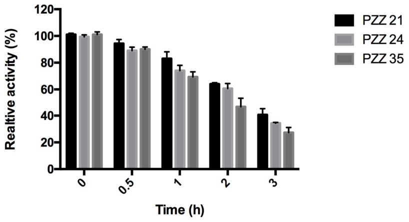

(b)

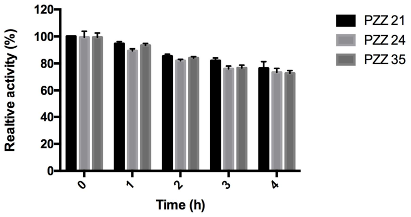

(c)

Figure S4. (**a**) Immunohistochemistry staining of scFvs in the jejunum sections of the pig. The section of jejunum were collected from the pig, sectioned at 10 μm thickness and stained with anti-His monoclonal antibody as primary antibody followed by FITC conjugated goat anti-mouse antibody as secondary antibody. The samples were visualized by fluorescence microscopy. (**b**) The stability of scFv to the simulated gastric conditions at pH 3. The scFv activity after incubation with SGF was assessed by ELISA and expressed as % activity relative to the scFv incubated with PBS. (**c**) The stability of scFv to the simulated intestinal conditions at pH 6.8. The scFv activity after incubation with SIF was assessed by ELISA and expressed as % activity relative to the scFv incubated with PBS.

Table S1. RT-PCR diagnosis of PEDV from clinical samples

Farms	Simple No.	specimen	Pig stage	Clinical Signs	PEDV	TGEV	PDcoV	Diagnosis
A	25	Fecal/small intestine	Suckling piglet	Diarrhea,dehydration, depression	21/25	0/25	0/25	PEDV
B	19	Fecal/small intestine	Suckling piglet	Diarrhea,dehydration, depression	15/19	0/19	0/19	PEDV
Sum	44				36/44			
